# Effects of a Gluten-Free Diet on Brain Bioelectrical Activity and Neurological Symptoms in Children with Celiac Disease: A Study Using EEG Assessment

**DOI:** 10.3390/jcm14030725

**Published:** 2025-01-23

**Authors:** Milena Zochowska-Sobaniec, Elzbieta Jarocka-Cyrta, Joanna Maria Lotowska, Piotr Sobaniec

**Affiliations:** 1 Department of Paediatric Neurology and Rehabilitation, Faculty of Health Sciences, Medical University of Bialystok, 15-274 Bialystok, Poland; milena.zochowska-sobaniec@umb.edu.pl; 2 Department of Developmental Age Medicine and Paediatric Nursing, Faculty of Health Sciences, Medical University of Bialystok, 15-295 Bialystok, Poland; 3 Neuromaster Institute of Neurophysiology, 15-068 Bialystok, Poland; 4 Department of Paediatrics, Gastroenterology and Nutrition, Faculty of Medicine, Collegium Medicum, University of Warmia and Mazury, 10-719 Olsztyn, Poland; elzbieta.jarocka@uwm.edu.pl; 5 Regional Specialized Children’s Hospital in Olsztyn, University of Warmia and Mazury, 10-561 Olsztyn, Poland; 6 Department of Medical Pathomorphology, Faculty of Medicine with the Division of Dentistry and Division of Medical Education in English, Medical University of Bialystok, 15-269 Bialystok, Poland; joanna.lotowska@umb.edu.pl

**Keywords:** celiac disease, EEG, gluten-free diet, neurological symptoms

## Abstract

Celiac disease (CeD), also known as gluten enteropathy, is an immune-mediated inflammatory enteropathy triggered by intolerance to gluten. It presents with a spectrum of symptoms, including both gastrointestinal and extraintestinal manifestations, as well as neurological symptoms. A review of the literature indicates that 10–22% of patients with CeD present with neurological symptoms. The objective of this study is to assess the influence of a gluten-free diet (GFD) on brain bioelectrical activity and neurological symptoms in children with CeD. **Methods:** The study was conducted using a multidisciplinary approach, encompassing a comprehensive array of clinical data gathered alongside laboratory test results, questionnaires, and electroencephalogram (EEG) assessments. The study population included 85 children: 18 newly diagnosed cases of CeD patients (NDC), subsequently reassessed after 6 months on a GFD as a celiac disease on diet (CDD); 27 CeD patients on a GFD for over 12 months (CDD2); and 40 healthy individuals in the comparison group (CG). **Results:** It was observed that over half of the NDC group exhibited neurological symptoms, particularly headaches. Following a six-month period on a GFD, there was a notable reduction in symptom severity. In comparison to the CG, the NDC patient group exhibited a higher prevalence of abnormalities in EEG recordings (*p* = 0.032), including focal sharp waves or slow waves. **Conclusions:** The results demonstrate that a GFD has a positive impact on the neurological condition of children with CeD. The clinical improvements correspond with EEG normalization, which supports the hypothesis that dietary intervention plays a role in mitigating CeD-associated neurological dysfunction.

## 1. Introduction

### 1.1. CeD

Celiac disease (CeD) represents one of the most prevalent autoimmune disorders [1]. The condition is triggered by a permanent intolerance to gluten, a storage protein found in grains such as wheat, rye, and barley [2]. It is estimated that the prevalence of CeD in the general population is between 1–3%. In Poland, approximately 1 in 160 people are affected by this condition [3]. Over the past decade, there has been an observed increase in the number of diagnosed cases of CeD in Western countries. This is partly a result of improved and more accessible diagnostic methods and greater awareness of the disease [4].

The clinical manifestation of CeD is markedly variable, exhibiting significant alterations with advancing age. In young children, the classic form of the disease is most prevalent, presenting with typical gastrointestinal symptoms related to malabsorption, including abdominal pain, bloating, chronic diarrhea, steatorrhea, weight loss, and growth retardation [5,6]. Genetic, immunological, and environmental factors also play an important role, although malabsorption of essential nutrients is a major risk factor for several CeD-associated morbidities [7]. In older patients, atypical symptoms are more prevalent, including neurological and psychiatric disorders, thyroid dysfunction, menstrual and pubertal disorders, bone mineralization abnormalities, enamel hypoplasia, chronic fatigue, dermatitis herpetiformis (also known as Duhring’s disease), and iron-deficiency anemia [8]. The diagnosis of CeD is based on three main categories of tests: serological, genetic, and histological. Serological tests are used to detect antibodies specific to the disease, while genetic tests determine the presence of human leukocyte antigen (HLA)-DQ2/DQ8. Histological evaluation is used to examine the damage caused by the disease in the small intestine [9,10].

The current diagnostic criteria for CeD are in accordance with the 2020 guidelines set forth by the European Society for Pediatric Gastroenterology, Hepatology, and Nutrition (ESPGHAN) [11]. According to the guidelines, the primary screening test performed in the pediatric population when visceral disease is suspected is the determination of anti-tissue transglutaminase 2 (anti-TG2) antibodies in immunoglobulin A (IgA) class and total IgA concentration. In the event that a patient is found to be IgA deficient, the next step is to determine anti-TG2, deamidated gliadin peptide (DGP), or endomysial antibody (EMA) levels in the immunoglobulin G (IgG) class. In cases where the anti-TG2 antibody concentration is 10 times above the upper limit of normal (ULN), a second blood draw is required for confirmation, and the determination of EMA titers is essential. The presence of positive test results (anti-TG2 10× > ULN and (+) EMA) substantiates a diagnosis of visceral disease, obviating the necessity for additional procedures such as a small bowel biopsy or genetic testing [11]. Conversely, when anti-TG2 antibody levels are less than 10 times the ULN, and in the presence of IgA deficiency and IgG class antibodies, histological evaluation of small intestinal mucosal sections should always be performed. In circumstances where CeD-specific antibodies (anti-TG2 and EMA) are detected, yet the histological evaluation of the duodenal mucosa reveals a normal, graded Marsh 0, or a diagnosis of elevated intraepithelial lymphocytosis (Marsh 1), genetic testing should be conducted to ascertain the presence of the HLA-DQ2/DQ8 haplotype [11,12]. In the 2012 ESPGHAN guidelines, it was permissible to diagnose CeD without a small bowel biopsy in children and adolescents with symptoms suggestive of the disease [13]. ESPGHAN expanded this group in 2020 to include patients without symptoms but from at-risk groups who are screened [11]. A notable distinction between the 2012 and 2020 guidelines pertains to the diagnostic approach for CeD without endoscopy. Prior to the 2020 update, the ESPGHAN guidelines mandated the use of EMA and HLA-DQ2/DQ8 haplotype determination as confirmation tests [13]. However, subsequent studies have demonstrated that these genetic tests do not enhance diagnostic accuracy, thus eliminating the necessity for their implementation [14]. Furthermore, recent guidelines recommend against evaluating DGP-IgA antibodies in children younger than two years of age, as their diagnostic utility for visceral disease is minimal [11].

The primary treatment modality is a strict, lifelong GFD [15]. In accordance with the guidelines set forth by the Food and Agriculture Organization of the United Nations (FAO/WHO), gluten-free products are required to contain less than 20 ppm (20 mg/kg) of gluten [16]. The clinical symptoms typically abate within a period of 2–3 weeks following the commencement of a GFD, while histological improvement is observed after a further 6–8 weeks [17]. A secondary indication of adherence to the GFD is the absence of celiac-specific antibodies, specifically anti-TG2 and EMA, in the blood serum. The initial follow-up serological test should be conducted six months after initiating the GFD to evaluate adherence. Subsequently, antibody levels should be measured annually in asymptomatic patients and whenever symptoms recur [18].

### 1.2. Neurological Symptoms and Psychological Dysfunction in CeD

As indicated in the extant literature, neurological and psychological disorders are observed in a prevalence of 10–22% among individuals diagnosed with CeD. These primarily affect adults, although they can also occur in children. Neurological manifestations include spinocerebellar ataxia, epilepsy, sensorineural hearing loss, neuropathy, myopathy, and headaches, as well as autism and ADHD. Additionally, a range of neuropsychiatric symptoms has been described, including sleep disturbances, depression, anxiety, and chronic fatigue [19,20,21].

Neurological symptoms may be the primary or sole manifestation of CeD. Due to the absence of typical gastrointestinal symptoms, neuroceliac disease may remain undiagnosed [22].

### 1.3. EEG Studies

Electroencephalography (EEG) is a research method utilized to assess the functionality of the central nervous system. The process entails the recording of the brain’s bioelectrical activity through the placement of electrodes on the scalp. In qualitative EEG analysis, the neurophysiologist employs a visual assessment of characteristics such as the background, dominant rhythm, spatial differentiation, maturation features, and reactivity, as well as the presence of epileptiform graphoelements and periodic or rhythmic patterns [23]. Resting-state EEG recordings provide insights into spontaneous neural activity, which is crucial for evaluating baseline brain function [24].

### 1.4. Aims and Objectives

Autoimmune mechanisms in CeD may result in functional impairment of the central nervous system in pediatric subjects. A review of the literature reveals a paucity of publications that address the qualitative assessment of EEG recordings in children with CeD. The results of these limited studies are inconclusive.

The objective of this study was to evaluate the influence of a GFD on the neurological and psychiatric symptoms, and brain bioelectrical activity in children with CeD.

## 2. Patients and Methods

### 2.1. Patients

The study was conducted at the Department of Paediatrics, Gastroenterology, and Paediatric Allergology at the Medical University of Bialystok Children’s Clinical Hospital, as well as the Department of Paediatric Neurology and Rehabilitation and the Institute of Neurophysiology Neuromaster in Bialystok. The study was approved by the Bioethics Committee of the Medical University of Bialystok (approval code: R-I-002/185/2013; date of approval: 25 April 2013).

The study cohort comprised 85 individuals, characterized in Table 1.

The study included 45 children with CeD and 40 healthy children who served as the comparison group (CG). Of these, 18 were patients with newly diagnosed CeD (NDC) without dietary intervention prior to initiating a GFD. Following a six-month period of diet adherence, the group was renamed CDD (celiac disease on diet) during the subsequent follow-up phase. The other group included 27 patients with celiac disease on diet 2 (CDD2), who had been on a GFD for a minimum of 12 months.

In order to be considered for inclusion in the study, participants were required to meet the following criteria:

(1) a diagnosis of CeD (ICD10: K90.0) according to ESPGHAN criteria [11];
(2) cooperation during EEG assessment;
(3) parental/guardian consent for participation in the study.

The exclusion criteria included the use of neurological and/or psychiatric medications and a history of traumatic brain injury. A history of epilepsy or seizures was not present among the celiac patients.

The CG consisted of patients without CeD, matched by sex and age, who followed a general diet and were excluded from CeD based on negative serological tests. They did not have any diagnosed neurological or psychiatric disorders and were not taking any medication for other conditions. In addition to the previously mentioned exclusion criteria, the following were also applied: lack of parental consent, a history of head injury, and inability to complete EEG recordings. Laboratory tests excluded the presence of anemia, electrolyte imbalance, iron deficiency, vitamin B12 deficiency, and folate deficiency.

The following procedures were undertaken for all participants in this study:

(1) initial qualification of participants;
(2) taking medical history, analysis of medical records, completion of surveys, and qualification for further stages;
(3) laboratory tests;
(4) EEG recordings.

No abnormalities were identified in the neurological examinations of any patients in the study or CG. Patients with headaches underwent central nervous system magnetic resonance imaging (MRI), which showed no significant abnormalities in the brain structures.

Each patient experiencing headaches had their blood pressure measured, and all recorded values were found to be below the 90th percentile for gender, age, and height. Patients were also asked to maintain a diary in which they recorded the characteristics of their headaches. The diaries were designed to record various details regarding the headaches, including the frequency, characteristics, severity, duration, location, precipitating factors, and associated symptoms.

In the cohort of patients with CeD on a GFD for a minimum of 12 months (CDD2), data pertaining to disease symptoms at the time of diagnosis and test results were retrospectively analyzed based on medical records.

**Figure 1:**
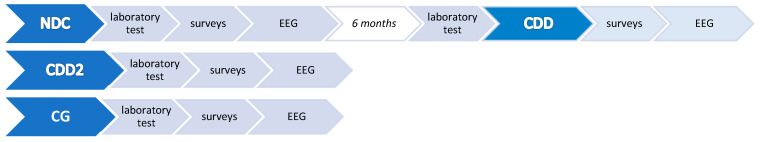
Study protocol, divided by groups and study stages. Definition of abbreviations: NDC = a newly diagnosed celiac group, CDD = celiac disease on diet group, CDD2 = celiac disease on diet for more than 12 months group, CG = comparison group, EEG = electroencephalography.

The study protocol is illustrated in Figure 1.

### 2.2. Methods

#### 2.2.1. Questionnaires

The study utilized a customized questionnaire completed by the children’s caregivers to assess a range of factors, including prenatal and neonatal periods, psychomotor development, symptoms associated with CeD, neurological and psychiatric disorders, and the presence and characteristics of headaches. The questionnaire included a series of questions pertaining to neurological symptoms, such as the occurrence of seizures, headaches, paresthesias, vision disturbances, gait and balance disorders, and abnormal muscle tone. In the context of this investigation, psychological dysfunction was defined as any deviation from the expected norm of cognitive, affective, or psychomotor processes. These included tics, memory problems, difficulties in concentration and attention, learning problems, irritability, apathy, low mood, and disturbances in the quality or patterns of sleep.

Patients experiencing headaches were requested to complete an additional questionnaire comprising questions regarding the frequency, location, intensity, duration, and nature of the headache, associated symptoms, triggering factors, and the impact of a GFD on reported symptoms.

To assess the intensity of headaches in children over the age of nine, the Numerical Rating Scale (NRS) was employed [25]. In younger children, the Wong-Baker FACES Pain Rating Scale was utilized [26].

The questionnaires asked about the clinical manifestations of CeD prior to diagnosis and the initiation of the GFD in a lifetime observation. Patients on a GFD diet were also asked whether the introduction of this diet resulted in a decrease in the severity and/or frequency of these complaints.

#### 2.2.2. Laboratory Tests

For each patient, the following were measured: anti-tissue transglutaminase 2 (TG2) antibody titers, complete blood count, iron homeostasis parameters (Fe, ferritin, UIBC), folate, vitamin B12, and electrolytes (Na, K, Cl, Mg, P, Ca).

#### 2.2.3. EEG Recordings

EEG signals were recorded using the BrainMaster Discovery 24E device (BrainMaster Technologies, Inc., Bedford, OH, USA) with ElectroCap (Electro-Cap International, Inc., Bedford, OH, USA) caps in six different sizes, selected based on head circumference from 48 to 58 cm. EEG signals were recorded from 19 electrodes placed on the scalp in accordance with the international 10–20 system. The electrodes utilized were Fp1, Fp2, F3, F4, C3, C4, P3, P4, O1, O2, F7, F8, T3, T4, T5, T6, Fz, Cz, Pz, and two ear electrodes, A1 and A2. The ground electrode (GND) in the ElectroCap is positioned on the FCz location. The EEG electrodes were filled with electro-gel, and the ear electrodes were applied with conductive adhesive the Ten20 paste. The impedance of all electrodes was below 10 kΩ. EEG signals were sampled at 256 Hz/s, with the EEG amplifier set to a passband of 0.5–70 Hz, and a 50 Hz notch filter.

The EEG was recorded for a minimum duration of 20 min in the morning while patients were in a supine position after a meal. The recording encompassed components of a standard resting-state EEG study, including recordings with eyes closed, reactivity to photostimulation, hyperventilation, and a comparison between eyes open and eyes closed.

The EEGs were identified as normal or within normal limits for age, indicating the preservation of age-specific characteristics, such as a posterior dominant rhythm with the anticipated frequency and spatially differentiated characteristics. EEG recordings exhibiting rare or occasional findings, such as focal sharp waves, sharp slow waves, or slow waves, were categorized as abnormal. Conversely, EEGs exhibiting paroxysmal or generalized epileptiform findings, including periodic discharges, rhythmic delta activity, epileptiform bursts, spike-wave discharges, or sharp-wave discharges, were classified as abnormal [27].

#### 2.2.4. Statistical Methods

The analysis of the study results was conducted using frequency analysis (percentage, n). For continuous variables, basic statistical measures such as the mean ± standard deviation (M ± SD) and the median (Me) were calculated, depending on the distribution. The relationship between two categorical variables was analyzed using Pearson’s chi-squared tests, and correlations between two continuous or ordinal variables were assessed using Spearman’s correlation analysis. To compare the values of continuous parameters between two groups, Student’s t-tests for independent samples were employed, or Mann–Whitney tests were utilized if the assumptions for parametric tests were not met. In cases where comparisons were to be made between more than two independent groups, an ANOVA was performed. In order to compare two dependent groups, Student’s t-tests for dependent samples or Wilcoxon tests were employed for continuous variables, while the Cochran test was utilized for nominal variables. A significance level of α = 0.05 was adopted, with the following conventions: * *p* < 0.05; ** *p* < 0.01; *** *p* < 0.001. The statistical analysis was performed using Statistica 13.3.

## 3. Results

### 3.1. CeD Symptoms

In the cohort of pediatric patients with CeD, the classical symptoms were the primary indications for the commencement of diagnostic procedures. The symptoms reported by the subjects included abdominal pain (27 individuals, 60%), chronic diarrhea (13 patients, 28.9%), low body weight (12 patients, 26.7%), and anemia (9 patients, 20%). Other symptoms included abdominal bloating in eight patients, nausea/vomiting in five patients, growth deficiency in five patients, and constipation in three patients. Subclinical CeD was diagnosed in five individuals who were classified as CDD2 patients. According to the medical records, the diagnosis of CeD in these children was made based on the 2012 ESPGHAN criteria, as outlined in the “Asymptomatic person at genetic risk for CD” algorithm [13]. The children in this group were from high-risk populations, with three having siblings diagnosed with CeD and two diagnosed with type 1 diabetes.

### 3.2. Laboratory Tests

Laboratory tests (Table 2) revealed the presence of mild microcytic anemia, accompanied by reduced serum iron and ferritin levels, in four patients who had recently been diagnosed with CeD. No folate deficiency or electrolyte imbalance was identified in any patient. One patient from the CDD group exhibited vitamin B12 levels that were below the lower limit of the normal range. Anti-TG2 antibody titers were elevated in all patients who had recently been diagnosed with CeD. Six months after the commencement of the GFD, anti-TG2 antibody levels remained elevated in six patients, exhibiting a tendency towards normalization. In all patients in the CDD2 and the CG, laboratory results were within the normal range, and no elevated anti-TG2 antibody levels were observed.

### 3.3. Neurological Symptoms and Psychological Dysfunction

**Figure 2:**
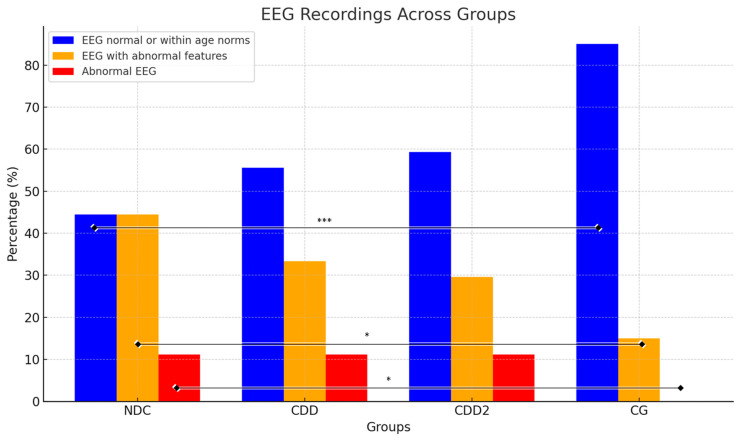
Qualitative assessment of EEG recordings in patients with celiac disease. A significant difference was observed between the NDC and CG groups for EEG recordings with normal (*p* < 0.001 ***), EEG with abnormal features (*p* = 0.017 *), and abnormal EEG (*p* = 0.032 *). Definition of abbreviations: NDC = a newly diagnosed celiac group, CDD = celiac disease on diet group, CDD2 = celiac disease on diet for more than 12 months group, CG = comparison group. Levels of significance: * *p* < 0.05, *** *p* < 0.001.

In the cohort of celiac patients (N = 45), 28 individuals (62.2%) reported the presence of neurological and psychological symptoms (Table 3, Figure 2).

The most prevalent symptom was headache, which was reported by twenty-five individuals (55.6%). Additional reported issues included difficulties with concentration and attention in ten individuals, irritability in ten individuals, sleep disturbances in six individuals, apathy in six individuals, learning difficulties in five individuals, low mood in five individuals, tics in four individuals, memory impairment in three individuals, and paresthesias in three individuals.

In the group of newly diagnosed celiac patients (NDC, N = 18), twelve individuals (66.7%) reported neurological symptoms, while nine individuals (50%) reported psychological dysfunction (Figure 2). A majority of patients (75%) reported at least two symptoms. The most prevalent neurological symptom was headaches, which were reported by twelve individuals. Additionally, one patient reported sensory disturbances in the form of limb numbness and tingling.

The psychological symptoms observed in this group included irritability in five individuals, concentration, and attention difficulties in five individuals, sleep disturbances in three individuals, learning difficulties in three individuals, tics in two individuals, apathy in two individuals, memory impairment in two individuals, and low mood in one individual.

At the six-month follow-up, psychological symptoms had improved in five individuals (27.8%), with three patients exhibiting full resolution of their symptoms. Notable improvements were observed in the following symptoms: irritability (a statistically significant reduction, *p* = 0.049 *), concentration and attention difficulties, sleep disturbances, memory problems, and learning difficulties. The number of individuals reporting neurological symptoms remained unchanged. No new symptoms were observed in any of the patients.

In the cohort of children who had been on a GFD for a longer duration (CDD2), 13 individuals (48.1%) reported neurological symptoms, while 11 individuals (40.7%) reported psychological dysfunction. A minimum of two symptoms were reported by 54% of the participants.

The most prevalent neurological disorder was headaches, which were reported by 13 individuals (48.1% of patients). Furthermore, two individuals exhibited paresthesia. The psychological symptoms observed in this group included irritability in five individuals, concentration and attention difficulties in five individuals, apathy in four individuals, low mood in four individuals, sleep disturbances in three individuals, learning difficulties in two individuals, tics in two individuals, and memory impairment in one individual.

In the CG, psychological dysfunction was reported by seven individuals (17.5%). A total of 85% of the individuals in this group reported two or more symptoms. These included difficulties with concentration and attention in five individuals, irritability in five individuals, learning difficulties in three individuals, sleep disturbances in two individuals, and memory impairment in one individual. No neurological symptoms were observed in any child from the CG, as the presence of such symptoms constituted an exclusion criterion for participation in the study.

In the NDC group, compared to the CG, there was a statistically significantly higher frequency of psychological symptoms (*p* = 0.009 **), tics (*p* = 0.032 *), and apathy (*p* = 0.032 *).

The Table 4 presents a comparative analysis of the incidence of histopathological changes according to the Marsh–Oberhuber classification in patients with CeD and neurological/psychological symptoms (N = 27) and those without neurological/psychological symptoms (N = 16). It should be noted that two patients with CeD who did not undergo a small bowel biopsy were excluded from this analysis.

### 3.4. EEG Studies

In newly diagnosed celiac patients (NDC), the EEG recording was normal in eight individuals (44.4%), abnormal in two individuals, and showed abnormal features in eight individuals (44.4%) (Table 5).

The prevalence of EEG abnormalities is significantly higher in the clinical groups (NDC, CDD, CDD2) compared to the CG. The consumption of gluten by newly diagnosed celiac NDC patients is associated with a high prevalence of EEG abnormalities, with approximately 55% of cases exhibiting such abnormalities. A six-month GFD of the CDD group has been demonstrated to improve EEG outcomes, with a 10% increase in the percentage of normal EEGs and a reduction in the prevalence of abnormal EEG features. Adherence to a GFD for a period of twelve months or longer has been demonstrated to result in a further enhancement in the normalization of EEGs in 60% of cases in the CDD2 group. Nevertheless, a subset of patients (12%) still exhibits abnormal EEGs, indicating the possibility of residual neurological dysfunction or incomplete recovery. In the CG, 85% of EEGs were normal, and no participants exhibited abnormal EEGs. This serves as a benchmark, indicating that the prevalence of EEG abnormalities in patients with CeD is notably higher than in the general population.

The hyperventilation reactivity of a single subject was documented. Three individuals exhibited photosensitivity to the intermittent photic stimulation, manifested as localized sharp waves. Two individuals exhibited paroxysmal changes, including one with a tendency for generalization. These included the occurrence of sharp wave–slow wave complexes in one patient and paroxysmal slow activity in another. The occurrence of sharp waves was documented in six individuals. Of these, two exhibited unilateral sharp waves, four demonstrated bilateral sharp waves, and in three cases, unilateral dominance was observed. Localization of the phenomena was observed in the posterior temporal-parietal-occipital areas in four individuals, in the parietal-occipital areas in one individual, and in the central-parietal areas in one individual.

Slow waves were documented in seven individuals, with unilateral occurrence in two cases and bilateral occurrence in five cases, exhibiting unilateral dominance in two cases. These included delta waves (less than 4 Hz) in one individual, theta waves (4–8 Hz) in two individuals, and co-occurring delta and theta waves in four individuals. Localization was observed in the posterior temporal-parietal-occipital areas in two individuals, the frontal-central areas in one individual, the anterior temporal-frontal areas in one individual, the frontal areas in one individual, the temporal areas in one individual, and the central-parietal-temporal areas in one individual.

Spindling Beta waves were documented in two subjects.

In the follow-up study, conducted six months after the commencement of the GFD, EEG improvement was observed in three of the ten individuals (30%) whose initial EEG was classified as abnormal or exhibiting abnormal features. Two individuals exhibited complete normalization of the EEG, while one individual demonstrated partial improvement. The differences were related to a reduction in the incidence, amplitude, or topography of the documented abnormalities.

In Patient I, the previously recorded sharp waves were absent in the bilateral posterior temporal-parietal-occipital areas, with right-sided dominance.

In the case of Patient II, the previously recorded sharp waves were no longer present in the bilateral parietal-occipital areas. Additionally, delta-theta slow waves (3–5 Hz) were observed in the bilateral parietal areas with right-sided dominance.

In Patient III, the absence of previously recorded theta focal slow waves (4–5 Hz) in the right temporal areas.

No deterioration in the EEG recordings was observed in any patient included in the study.

In the cohort of celiac patients on a GFD (CDD2), a normal electroencephalogram (EEG) was recorded in 16 individuals (59.3%). Abnormal EEG recordings were identified in three individuals (11.1%), and EEGs with abnormal features were documented in eight individuals (29.6%). One subject demonstrated hyperventilation reactivity, while another exhibited photosensitivity. Two individuals exhibited paroxysmal changes, including one with a tendency for generalization. These included the occurrence of sharp wave–slow wave complexes, spike–wave complexes in one individual, and paroxysmal slow waves in one individual. Unilateral sharp waves were recorded in one individual, while bilateral sharp waves were recorded in five individuals, with unilateral dominance in four of those individuals. Localization of the abnormalities was observed in the following areas: one individual exhibited abnormalities in the posterior temporal-parietal-occipital areas, one in the parietal-occipital areas, one in the central-parietal-temporal areas, one in the frontal-central-parietal areas, one in the posterior temporal-occipital areas, and one in the frontal-anterior temporal areas. Slow waves were recorded bilaterally in six individuals. These included delta waves with a frequency of less than 4 Hz in one individual, theta waves with a frequency of 4 to 8 Hz in two individuals, and co-occurring delta and theta waves in three individuals. Localization was observed in the frontal-central areas in three individuals, the frontal-central-anterior temporal areas in one individual, the posterior temporal-parietal-occipital areas in one individual, and the frontal-anterior temporal areas in one individual.

In the CG, EEG recordings were within the normal range in 34 individuals (85%). Six individuals (15%) exhibited EEG abnormalities. No significant reactivity to hyperventilation or photostimulation was observed. No paroxysmal or generalized changes were observed. Unilateral sharp waves were observed in one subject, while bilateral sharp waves were recorded in three subjects, without clear lateralization dominance. Localization was observed in the posterior temporal-parietal areas in one individual, the parietal-occipital region in two individuals, and the central-parietal region in one individual. Slow waves were recorded in three individuals, with one exhibiting unilateral activity and the other two demonstrating bilateral activity with unilateral dominance. Additionally, two individuals exhibited co-occurring delta waves (less than 4 Hz) and theta waves (4–8 Hz), while one individual demonstrated theta waves exclusively. Localization was observed in the frontal-central areas in one individual, the central-parietal areas in one individual, and the posterior temporal-parietal areas in one individual.

Spindling Beta waves were documented in a single subject.

In the NDC group, compared to the CG, there was a statistically significantly higher frequency of the following: The EEGs with abnormal features (*p* = 0.017), the EEGs with abnormalities (*p* = 0.032), the paroxysmal changes (*p* = 0.032), the photosensitivity (*p* = 0.007), the sharp waves (*p* = 0.029), and the sharp waves in temporal areas (*p* = 0.012) were found to be statistically significantly different between the two groups. Additionally, there were statistically significant differences in the occurrence of sharp waves in parietal areas (*p* = 0.029), sharp waves in occipital areas (*p* = 0.013), slow waves (*p* = 0.0028), slow waves in temporal areas (*p* = 0.0029), and slow waves in occipital areas (*p* = 0.032).

No statistically significant differences were identified between the CDD-CDD2 and NDC-CDD2 groups. In the NDC group, a positive correlation was observed between anti-TG2 titers and the occurrence of EEG abnormalities (*p* = 0.014, r = 0.57).

## 4. Discussion

The extant evidence indicates that immunological processes exert a preponderant influence on the pathogenesis of neurocardiac disease. A disruption of the blood–brain barrier may be the underlying cause of certain neurological disorders. The increased permeability of the blood–brain barrier in CeD may be attributed to the deposition of immune complexes in the brain’s blood vessels, perivascular inflammatory cell infiltrates, and direct cytotoxicity of T lymphocytes [28,29]. Additionally, neurological disorders may be caused by the presence of autoantibodies formed through molecular mimicry and cross-reactivity.

The results of studies on the pathomechanism of gluten-related ataxia indicate the potential for cross-reactivity of antibodies between antigenic epitopes on Purkinje cells in the cerebellum and gliadin. In the case of gluten neuropathy, there is evidence indicating cross-reactivity of antigliadin antibodies with a neuronal protein, synapsin I. Additionally, it has been demonstrated that gliadin fragments, following glycosylation, may exhibit structural similarities to ganglioside epitopes in nerve cells. Antigliadin antibodies have the capacity to bind to ganglioside epitopes, thereby triggering an autoimmune response against nerve cells [30,31].

The observed neurological symptoms in CeD may also result from secondary vitamin and nutrient deficiencies due to malabsorption syndrome. This condition, characterized by the inability to properly digest and absorb nutrients from food, can also contribute to low blood glucose levels. Hypoglycemia can be a significant factor in causing headaches. According to the International Classification of Headache Disorders (ICHD-3), the group of secondary or symptomatic headaches includes complaints related to disorders of homeostasis [32]. Among them we distinguish fasting headache that is headache triggered by fasting. It is believed that even small fluctuations in blood glucose levels, especially if they persist for a long time, can cause headaches [33]. It is possible that improvements in gastrointestinal function and carbohydrate metabolism, which normalize blood glucose, may be responsible for reduction in headaches after gluten elimination in celiac patients.

Nevertheless, research findings suggest that nutritional deficiencies resulting from gluten intolerance do not solely account for the observed neurological symptoms. This is demonstrated by instances of neuroceliac disease in which there are no clinical indications of malabsorption syndrome, as well as observations in which the supplementation of appropriate vitamins and micronutrients did not result in symptom resolution [34].

### 4.1. Neurological Disorders in CeD

Adults with CeD are more susceptible to developing neurological and psychiatric disorders compared to children. The prevalence of neuroceliac disease in adults is 26%, while in children, it is 2.6% [35].

Neurological symptoms that manifest in adult patients with CeD are exceedingly rare in the pediatric population. Such examples include cerebellar ataxia and neuropathy. The relative protection of children from neurological complications may be attributed to their shorter exposure to immunogenic gluten peptides.

The developing nervous system is less susceptible to harmful factors, and a requisite period of time must elapse for pathogenic antibodies to penetrate the central nervous system and cause irreversible damage. The early diagnosis of CeD in childhood allows for the earlier initiation of dietary therapy, which may protect against the development of neurological disorders. Observational studies have demonstrated that children adhere to a GFD with greater compliance than adolescents and adults [36]. According to Jericho et al., a GFD is significantly more effective in alleviating extraintestinal symptoms in children with CeD compared to adults. Lack of improvement may also indicate more frequent dietary errors or the presence of comorbidities [36].

In a study conducted by Isikay et al., which included a large cohort of children with CeD, 13.5% of the participants exhibited neurological symptoms. These included non-specific headaches (9.7%), migraine (4.4%), and epilepsy (5.4%), with generalized seizures occurring in 62.5% and partial seizures in 37.5% of cases. Additionally, intellectual disability (4%), ataxia (0.7%), and ADHD (0.7%) were also documented in the literature [37].

In our study, at the time of the diagnosis of CeD, the majority of patients exhibited neurological symptoms of varying severity. The prevalence of these symptoms in children newly diagnosed with CeD was 66.7%. As observed in the aforementioned study by Isikay et al., the most prevalent symptom was headache [38]. Additionally, a small number of patients reported paresthesias. However, none of the examined patients experienced seizures, abnormal muscle tone, visual disturbances, gait, or balance disorders described by Isikay et al. The discrepancies in the obtained data may be attributed to the varying sample sizes in both studies.

No statistically significant differences were observed in laboratory test results, including hemoglobin, mean corpuscular volume (MCV), iron, ferritin, vitamin B12, folic acid, alanine transaminase (ALT), aspartate transaminase (AST), alkaline phosphatase (ALP), calcium, phosphorus, vitamin D, thyroid stimulating hormone (TSH), thyroxine, or albumin, between individuals with and without CeD experiencing neurological problems, as reported by Isikay et al. [38]. These findings are in accordance with our results. The present study did not find a correlation between the presence of neurological symptoms and abnormal laboratory findings.

A comparison of the histopathological assessment of biopsies revealed that the occurrence of neurological complications was dependent on the severity of enteropathy. Type 3a, according to the Marsh–Oberhuber classification, was more prevalent in patients without neurological disorders (*p* = 0.001), whereas type 3b was statistically significantly more common in patients with neurological disorders (*p* < 0.001) [38]. The results of this study indicate that, upon histopathological evaluation of duodenal biopsies, type 3c was more prevalent in patients with neurological disorders. Conversely, type 3a and 3b were more frequently observed in children with CeD without neurological disorders. However, these differences did not reach statistical significance.

Dietary adherence in the study groups was evaluated through interviews with parents, analysis of dietary logs, and serum anti-TG2 titers.

Six months after the introduction of a GFD, anti-TG2 titers remained elevated in 33.3% of patients, yet the values demonstrated a downward trend.

As evidenced in the literature, there is a considerable degree of variability in the time required for anti-TG2 titers to normalize. In both pediatric and adult patients, the process of achieving clinical remission may take a year or longer after the initiation of a GFD, during which the disease remains active [39]. In a study conducted by Isaac et al., the median time to normalization was 407 days for 80.5% of patients [40].

The prolonged time to normalize anti-TG2 titers may explain why CeD symptoms persist longer despite the implementation of a GFD at the time of diagnosis. In studies conducted by Majsiak et al., the longest-observed symptom in children diagnosed with CeD was headaches, with an average duration of 5.5 years [41]. At the six-month mark of the GFD, 66.7% of individuals continued to experience headaches, although 58.3% demonstrated some degree of symptom alleviation. Among the pediatric cohort who had been on a GFD for an extended period (an average of 3 years and 2 months), headaches persisted in 48.1% of patients.

### 4.2. Psychological Functioning Disorders in CeD

A multicenter study conducted by Butwicka et al. demonstrated that autoimmune gluten intolerance may increase the risk of developing psychiatric disorders in children. The results of the analysis indicated that children with CeD exhibited a 1.4-fold increased risk of developing psychiatric illness, including mood disorders, anxiety, eating disorders, conduct disorders, and intellectual disability, in comparison to the general population [42].

A meta-analysis conducted by Clappison et al., which included 37 publications on psychiatric disorders in CeD, demonstrated a significant increase in the risk of depression, anxiety, autism spectrum disorders, attention-deficit/hyperactivity disorder (ADHD), and eating disorders among celiac patients compared to a CG. No significant differences were observed with regard to schizophrenia and bipolar disorder [43].

In 2017, Pediatrics published a report from the TEDDY study group, which indicated a higher incidence of reports of anxiety, depression, aggressive behavior, and sleep problems among children with CeD compared to the CG [44].

Furthermore, this study demonstrated that children with CeD exhibited a significantly higher prevalence of psychological functioning disorders compared to the CG. Prior to the implementation of a GFD, the prevalence of psychiatric symptoms in children with CeD was 50%. Among the observed symptoms, those pertaining to concentration and attention, as well as irritability, were particularly prevalent. Additionally, the study group exhibited a range of other symptoms, including sleep disturbances, apathy, memory problems, learning difficulties, low mood, and tics. Long-term, strict adherence to a GFD for a period exceeding 12 months resulted in a reduction in the frequency of the aforementioned symptoms.

There is mounting evidence that CeD, when left untreated, also impairs cognitive function. These so-called “silent” neuropsychological complications include subtle impairments in memory, attention, concentration, decision-making, and cognitive processing speed. It is estimated that the prevalence of these disorders in celiac patients ranges from 2% to 10%. The highest prevalence is observed among late-diagnosed older adults and those who do not adhere to a GFD [45,46].

Adherence to a GFD may have social consequences, including isolation, avoidance of social gatherings and eating outside the home due to the risk of dietary mistakes, and the constant need to explain the diagnosis. Psychological support is crucial for fostering acceptance and mitigating the risk of anxiety and depression [47,48].

In the most recent studies, Canova et al. observed a significant improvement in quality of life, anxiety, and depressive symptoms one year after the diagnosis of CeD in patients adhering to a GFD. In the analysis conducted by Enaud et al., the primary factors associated with enhanced quality of life were adherence to a GFD and the length of time over which this diet was maintained. The authors posit that these findings may offset the restrictive nature of the diet and enhance its acceptance among patients [49].

### 4.3. EEG in CeD

As evidenced in the extant literature, more than 10% of individuals in good health may demonstrate non-specific abnormalities in EEG. It is estimated that approximately 1% of recorded EEGs may contain epileptiform changes. The prevalence of these abnormalities is higher in children, with figures ranging from 2% to 4% [50,51]. The range of EEG pathologies observed in individuals with CeD is extensive. The most common focal abnormalities are unilateral or bilateral spikes, sharp waves, or slow wave complexes, which are primarily localized in the occipital lobes [52,53].

Nevertheless, the literature underscores that EEG patterns should not be regarded as exclusive to this disease. Additionally, diffuse, frontal, temporal, and parietal changes have been documented [53]. In a study conducted by Isikay et al., epileptiform elements were identified in 9.3% of newly diagnosed celiac patients, 1.5% of previously diagnosed patients, and 1% of the CG. A statistically significant difference was identified between the groups with regard to the prevalence of EEG abnormalities. The presence of epileptiform activity was found to be positively correlated with serum anti-tissue transglutaminase antibodies [54,55]. Our findings corroborate this relationship. In patients with CeD, a positive correlation was observed between anti-TG2 titers and EEG-recorded abnormalities.

In our study, a statistically significantly higher prevalence of abnormal or within normal EEGs, in the form of paroxysmal changes and focal changes (sharp waves, slow waves of varying localization), was observed in children newly diagnosed with CeD compared to the CG. These observations are consistent with the findings of Parisi et al., who reported that 48% of children with CeD exhibited EEG abnormalities [56].

In this study, the temporal, parietal, and occipital regions were the most frequently affected areas in newly diagnosed celiac patients, as evidenced by changes recorded in EEG. This conclusion is partially consistent with the findings of Isikay et al., which indicated that the occipital and posterior-temporo-parieto-occipital regions on the left side were the most prevalent [55]. Similar results were reported by Parisi et al., who observed abnormalities in the frontal, parietal, and occipital regions, as well as generalized changes [56].

It is noteworthy that epileptiform changes may manifest in the context of neurological disorders that are not necessarily epileptic in nature. Such examples include spikes in the occipital regions in children with congenital visual impairments or paroxysmal sharp waves in patients with migraines. Additionally, episodic focal slow waves are non-specific. Such occurrences have been documented in both healthy individuals and patients presenting with conditions such as migraines, mild cerebrovascular disease, and even brain tumors [57].

Boutros et al. demonstrated a higher prevalence of isolated epileptiform elements in the EEG of patients with psychiatric disorders compared to the CG [58]. As evidenced in the literature, the prevalence of these abnormalities in EEG is notably elevated in children and adolescents diagnosed with ADHD [59], ASD [60,61,62], and depression [63].

Arns et al. employed EEG phenotype analysis to demonstrate that, in addition to the commonly described patterns associated with increased slow wave activity, mainly theta (which affected 47% of the study group), 22% of the study group also exhibited a phenotype of increased beta activity or spindling beta pattern. These findings correlated with increased hyperactivity, impulsivity, and inattention in the study group [64,65]. In the present study, a spindle beta pattern was identified in 11.1% of children who had recently been diagnosed with CeD, suggesting a potential association with previously reported psychological disorders, including irritability and sleep disturbances.

Swatzyna et al. conducted an analysis of a clinical database comprising diagnostic, demographic, EEG, and QEEG data from children aged 5–17 with a diverse range of psychiatric disorders and inadequate pharmacological treatment. In the patients’ EEGs, four types of non-specific abnormalities were identified: encephalopathies, focal slowing, epileptiform discharges, and spindle beta. The authors concluded that there is a statistically significant association between the presence of spindle beta and depressive disorders, although only 35% of individuals with spindle beta in their EEG were diagnosed with depression [66].

In this study, the impact of a GFD on EEG recordings in children with CeD was evaluated. At the six-month follow-up examination, a reduction in the amplitude or frequency of sharp waves and slow waves in various locations was observed in 30% of cases. This result is less pronounced than that reported by Parisi et al., who observed normalization of the recording in 77.7% of patients following six months of adherence to a GFD [56]. It seems unlikely that complete recording improvement will be achieved, and the discrepancies in the percentage results regarding improvement may be attributed to the varying sample sizes in the study groups.

The findings of Mavroudi et al. indicate that the efficacy of a GFD is inversely correlated with the duration of neurological disorders prior to its implementation, as well as the age at which gluten intolerance is diagnosed and treatment is initiated [67].

In a population-based cohort study published in 2020 in the European Journal of Neurology, the prevalence of epilepsy among children diagnosed with CeD was estimated at 2.6% [68]. It is notable that no epilepsy was diagnosed in any child with CeD in this study, which may be attributed to the relatively small sample size.

## 5. Conclusions

The findings of the study suggest that the elimination of gluten from the diet may have a beneficial effect on the neurological condition of children diagnosed with CeD. An improvement in patients’ clinical status was found to correlate with an improvement in qualitative electroencephalographic recordings. However, persistent abnormalities in some patients indicate the necessity of early diagnosis and long-term dietary compliance to optimize neurological outcomes.

## 6. Study Limitations

The present study was conducted with a few limitations, including a relatively small sample size, a single-center study design, and voluntary participation by parents, which could have introduced some degree of selection bias. Additionally, there is currently a paucity of studies in this area, which precludes a comparison of our findings with those of other studies. Therefore, further research on a larger number of individuals is needed to expand and verify the current findings of our study.

## Figures and Tables

**Table 1:** Characteristics of Patients.

		CeD (N = 45)	Comparison Group (N = 40)	*p*
Age (years)		10.5 ± 3.8	9.5 ± 3.5	0.264
Gender (n)	Female	30	25	0.80
	Male	15	15	1
GFD (n)	No	18 (NDC)	40 (CG)	-
	Yes	18 (CDD) 27 (CDD2)	0	-
Age of diagnosis of CeD (mean ± SD)	NDC	10.05 ± 4.39	-	NDC—CDD2 0.008 **
	CDD2	6.99 ± 3.04		
The duration of time on a GFD (year: mean ± SD)	CDD	0.61 ± 0.16	-	CDD—CDD2 0.003 **
	CDD2	3.15 ± 2.85		

Definition of abbreviations: CeD = Celiac disease, GFD = a gluten-free diet, NDC = a newly diagnosed celiac, CDD = celiac disease on diet, CDD2 = celiac disease on diet for more than 12 months, CG = comparison group. ** *p* < 0.01.

**Table 2:** Laboratory results in each study group (mean ± SD).

	NDC N = 18	CDD N = 18	CDD2 N = 27	CG N = 40	*p* NDC—CG	*p* NDC—CDD	*p* NDC—CDD2
anti-TG2	38.71 ± 30.56	8.77 ± 11.87	1.46 ± 1.23	0.94 ± 0.87	<0.001 ***	<0.001 ***	<0.001 ***
RBC	4.57 ± 0.72	4.75 ± 0.55	4.78 ± 0.67	4.79 ± 0.46	0.071	0.083	0.069
HGB	12.66 ± 0.92	12.89 ± 0.35	13.02 ± 0.87	12.99 ± 0.65	0.136	0.354	0.392
HCT	36.58 ± 2.91	37.81 ± 1.62	38.13 ± 2.5	37.70 ± 1.68	0.197	0.129	0.069
MCV	76.95 ± 9.11	78.04 ± 6.77	80.08 ± 4.46	80.35 ± 3.85	0.156	0.686	0.190
Ferritin	34 ± 19.11	45.08 ± 13.19	46.85 ± 17.93	50.02 ± 13.85	0.004 **	0.037*	0.027 *
Fe	73.88 ± 21.5	76.94 ± 19.06	93.85 ± 26.63	89.48 ± 19.31	0.005 **	0.037*	0.013 *
UIBC	259.06 ± 75.87	256.83 ± 39.52	247.81 ± 40.4	243.38 ± 33.97	0.668	0.062	0.459
folic acid	10.56 ± 2.73	10.87 ± 2.58	9.3 ± 2.6	10.88 ± 2.12	0.459	0.782	0.123
Vitamin B12	313.77 ± 57.9	330.17 ± 47.99	381.21 ± 100.15	354.13 ± 80.10	0.036 *	0.361	0.007 **

There was a statistically significant difference between NDC and patients on a GFD and the comparison group in terms of anti-TG2, ferritin, iron, and vitamin B12 concentrations. Patients diagnosed with CeD before the introduction of the GFD had lower ferritin, iron, and vitamin B12 levels compared to patients on the GFD for more than 12 months (*p* = 0.027 *; *p* = 0.013 *; *p* = 0.007 **, respectively) and patients in the CG (*p* = 0.004 **; *p* = 0.005 **; *p* = 0.036 *, respectively). After 6 months on a GFD (CDD), there was a statistically significant increase in ferritin and iron levels (*p* = 0.037 * and *p* = 0.037 *, respectively). It should be noted that vitamin B12 concentrations in each analyzed group were within normal limits, although the differences in these concentrations showed significant differences. Definition of abbreviations: anti-TG2 = anti-tissue transglutaminase 2 (TG2) antibody titers, CeD = celiac disease, GFD = a gluten-free diet, NDC = a newly diagnosed celiac group, CDD = celiac disease on diet group, CDD2 = celiac disease on diet for more than 12 months group, CG = comparison group. Data are expressed as mean ± SD. Levels of significance: * *p* < 0.05, ** *p* < 0.01, *** *p* < 0.001.

**Table 3:** Characteristics of the presence of neurological and psychological symptoms in each study group.

Symptoms	NDC N = 18		CDD N = 18		*p* (NDC—*CDD*)	Change NDC—CDD	CDD2 N = 27		CG N = 40		*p* (NDC—CG)	*p* (CDD2—CG)
	n	%	n	%			n	%	n	%		
Neurological symptoms	12	66.7%	12	66.7%	-	No change	13	48.1%	0	0%	-	-
Psychological symptoms	9	50%	6	33.3%	0.324	Improvement in 3 people	11	40.7%	7	17.5%	0.009 **	0.035 *
Headaches	12	66.7%	12	66.7%	-	No change	13	48.1%	0	0%	-	-
Tics	2	11.1%	2	11.1%	-	No change	2	7.4%	0	0%	0.032 *	0.082
Sleep disturbances	3	16.7%	2	11.1%	0.317	Improvement in 1 person	3	11.1%	2	5.0%	0.143	0.358
Irritability	5	27.8%	2	11.1%	0.049 *	Improvement in 3 people	5	18.5%	5	12.5%	0.154	0.505
Apathy	2	11.1%	2	11.1%	-	No change	4	14.8%	0	0%	0.032 *	0.011 *
Depressed mood	1	5.6%	1	5.6%	-	No change	4	14.8%	0	0%	0.133	0.011 *
Learning difficulties	3	16.7%	1	5.6%	0.157	Improvement in 2 people	2	7.4%	3	7.5%	0.288	0.988
Attention problems	5	27.8%	3	16.7%	0.157	Improvement in 2 people	5	18.5%	5	12.5%	0.154	0.505
Memory problems	2	11.1%	1	5.6%	0.317	Improvement in 1 person	1	3.7%	1	2.5%	0.171	0.780
Paresthesias	1	5.6%	1	5.6%	-	No change	2	7.4%	0	0%	0.133	0.082

A statistically significant decrease was observed for symptoms such as psychological symptoms, tics, irritability, and depressed mood, particularly when comparing the NDC and CDD groups to the CG. Additionally, there was a statistically significant improvement in irritability, with no significant change observed in learning difficulties, concentration, attention, or memory problems. Definition of abbreviations: NDC = a newly diagnosed celiac group, CDD = celiac disease on diet group, CDD2 = celiac disease on diet for more than 12 months group, CG = comparison group. Data are expressed as: n, % values. Levels of significance: * *p* < 0.05, ** *p* < 0.01.

**Table 4:** Histopathologic changes according to the Marsh–Oberhuber classification in the group of patients with celiac disease and neurological/psychological symptoms (N = 27) and without neurological/psychological symptoms (N = 16).

Histopathological Changes	Patients with Celiac Disease N = 43				
	Neurological/Psychological Symptoms Are Present N = 27		Neurological/Psychological Symptoms Absent N = 16		
	n	%	n	%	*p*
3a	4	14.8%	3	18.6%	0.723
3b	18	66.7%	11	68.8%	0.288
3c	5	18.5%	2	12.5%	0.505

**Table 5:** Qualitative evaluation of EEG recordings in the study groups.

Parameter	NCD		CDD		*p*	Change	CDD2		CG		*p*
	N = 18		N = 18		NCD—CDD	NCD—CDD	N = 27		N = 40		NCD—CG
	n	%	n	%			n	%	n	%	
Normal or within normal EEG	8	44.4%	10	55.6%	0.519	2 people. from ‘with abnormal features’ to ‘normal EEG’	16	59.3%	34	85.0%	0.00 1 ***
EEG with abnormal features	8	44.4%	6	33.3%	0.508		8	29.6%	6	15.0%	0.017 *
Abnormal EEG	2	11.1%	2	11.1%	1.000		3	11.1%	0	0.0%	0.032 *
Spatially differentiated	18	100.0%	18	100.0%	1.000	No change	27	100.0%	40	100.0%	-
The posterior dominant rhythm	18	100.0%	18	100.0%	1.000	No change	27	100.0%	40	100.0%	-
Eye closure sensitivity	18	100.0%	18	100.0%	1.000	No change	27	100.0%	40	100.0%	-
Focal, paroxysmal discharges	2	11.1%	2	11.1%	1.000	No change	2	7.4%	0	0.0%	0.032 *
Generalized discharges	1	5.6%	1	5.6%	1.000	No change	1	3.7%	0	0.0%	0.137
The response to hyperventilation	1	5.6%	1	5.6%	1.000	No change	1	3.7%	0	0.0%	0.137
Photosensitivity	3	16.7%	3	16.7%	1.000	No change	1	3.7%	0	0.0%	0.007 **
Sharp waves	6	33.3%	5	27.8%	0.723	Improving the record of 1 person	6	22.2%	4	10%	0.029 *
- unilaterally	2	11.1%	2	11.1%	1.000	No change	1	3.7%	1	2.5%	0.171
- bilaterally	4	22.2%	3	16.7%	0.681	Improving the record of 1 person	5	18.5%	3	7.5%	0.11
- bilateral with dominance of one side	3	16.7%	2	11.1%	0.681	Improving the record of 1 person	4	14.8%	0	0.0%	0.0074 **
- in frontal regions	0	0.0%	0	0.0%	-	No change	2	7.4%	0	0.0%	-
- in temporal regions	4	22.2%	3	16.7%	0.681	Improving the record of 1 person	4	14.8%	1	2.5%	0.012 *
- in central regions	1	5.6%	1	5.6%	1.000	No change	2	7.4%	1	2.5%	0.56
- in parietal regions	6	33.3%	4	22.2%	0.47	Improving the record in 2 people	4	14.8%	4	10%	0.029 *
- in occipital regions	5	27.8%	3	16.7%	0.43	Improving the record of 2 people	3	11.1%	2	5%	0.013 *
Slow waves	7	38.9%	6	33.3%	0.73	Improving the record of 1 person	6	22.2%	3	7.5%	0.0028 **
- delta	5	27.8%	5	27.8%	1.000	No change	4	14.8%	2	5%	0.013 *
- theta	6	33.3%	5	27.8%	0.72	Improving the record of 1 person	5	18.5%	3	7.5%	0.011*
- unilaterally	2	11.1%	1	5.6%	0.56	Improving the record of 1 person	0	0.0%	1	2.5%	0.17
- bilaterally	5	27.8%	5	27.8%	1.000	No change	6	22.2%	2	5%	0.013 *
- both sides with domination of one side	2	11.1%	2	11.1%	1.000	No change	2	7.4%	2	5%	0.032 *
- in frontal regions	3	16.7%	3	16.7%	1.000	No change	5	18.5%	1	2.5%	0.049 *
- in temporal regions	5	27.8%	4	22.2%	0.710	Improving the record of 1 person	3	11.1%	1	2.5%	0.0029 **
- in central regions	2	11.1%	2	11.1%	1.000	No change	4	14.8%	2	5%	0.40
- in parietal regions	3	16.7%	2	11.1%	0.641	Improving the record of 1 person	2	11.1%	2	5%	0.14
- in occipital discharges	2	11.1%	2	11.1%	1.000	No change	2	11.1%	0	0.0%	0.032 *
The sharp wave-slow wave discharges	1	5.6%	1	5.6%	1.000	No change	1	3.7%	0	0.0%	0.13
The spike-wave discharges	0	0.0%	0	0.0%	-	No change	1	3.7%	0	0.0%	-
The polyspike-wave discharges	0	0.0%	0	0.0%	-	No change	0	0.0%	0	0.0%	-
Spindling beta	2	11.1%	2	11.1%	1.000	No change	0	0.0%	1	2.5%	0.17

Definition of abbreviations: NDC = a newly diagnosed celiac group, CDD = celiac disease on diet group, CDD2 = celiac disease on diet for more than 12 months group, CG = comparison group. Levels of significance: * *p* < 0.05, ** *p* < 0.01, *** *p* < 0.001.

## Data Availability

The original contributions presented in this study are included in the article. Further inquiries can be directed to the corresponding author.
